# How parents can affect excessive spending of time on screen-based activities

**DOI:** 10.1186/1471-2458-14-1261

**Published:** 2014-12-12

**Authors:** Daniela Brindova, Jan Pavelka, Anna Ševčikova, Ivan Žežula, Jitse P van Dijk, Sijmen A Reijneveld, Andrea Madarasova Geckova

**Affiliations:** Health Psychology Unit, Institute of Public Health, Faculty of Medicine, P.J. Safarik University in Kosice, Tr. SNP 1, Košice, 041 01 Slovak Republic; Institute of Active Living, Faculty of Physical Culture, Palacky, University in Olomouc, Tr. Miru 115, Olomouc, 77111 Czech Republic; Center for Kinanthropology Research, Institute of Active Lifestyle, Faculty of Physical Culture, Palacky University in Olomouc, Tr. Miru 115, Olomouc, 77111 Czech Republic; Institute for Research of Children, Youth and Family, Faculty of Social Studies, Masaryk University in Brno, Joštova 10, 602 00 Brno, Czech Republic; Institute of Mathematics, Faculty of Natural Sciences, P.J. Safarik University in Kosice, Jesenná 5, 040 01 Košice, Slovakia; Olomouc University Social Health Institute (OUSHI), Palacký University in Olomouc, Univerzitní 22, 771 11 Olomouc, Czech Republic; Department of Community & Occupational Health, University Medical Center Groningen, University of Groningen, A. Deusinglaan 1, 9713 AV Groningen, The Netherlands

**Keywords:** Screen-based activities, Family-shared activities, Parental rules

## Abstract

**Background:**

The aim of this study is to explore the association between family-related factors and excessive time spent on screen-based activities among school-aged children.

**Methods:**

A cross-sectional survey using the methodology of the Health Behaviour in School-aged Children study was performed in 2013, with data collected from Slovak (n = 258) and Czech (n = 406) 11- and 15-year-old children. The effects of age, gender, availability of a TV or computer in the bedroom, parental rules on time spent watching TV or working on a computer, parental rules on the content of TV programmes and computer work and watching TV together with parents on excessive time spent with screen-based activities were explored using logistic regression models.

**Results:**

Two-thirds of respondents watch TV or play computer games at least two hours a day. Older children have a 1.80-times higher chance of excessive TV watching (CI: 1.30-2.51) and a 3.91-times higher chance of excessive computer use (CI: 2.82-5.43) in comparison with younger children. More than half of children have a TV (53%) and a computer (73%) available in their bedroom, which increases the chance of excessive TV watching by 1.59 times (CI: 1.17-2.16) and of computer use by 2.25 times (CI: 1.59-3.20). More than half of parents rarely or never apply rules on the length of TV watching (64%) or time spent on computer work (56%), and their children have a 1.76-times higher chance of excessive TV watching (CI: 1.26-2.46) and a 1.50-times greater chance of excessive computer use (CI: 1.07-2.08). A quarter of children reported that they are used to watching TV together with their parents every day, and these have a 1.84-times higher chance of excessive TV watching (1.25-2.70).

**Conclusions:**

Reducing time spent watching TV by applying parental rules or a parental role model might help prevent excessive time spent on screen-based activities.

## Background

New information and communication technologies have become an important part of adolescents’ everyday lives. More and more children use them not only as a supporting component in education, but also as a form of entertainment and use of leisure time. Research suggests that children are spending too much of their free time on screen-based activities, including playing PC games, using the Internet or watching TV [1]. According to findings of the international HBSC study, around 60% of 11- and 15-year-old adolescents watch television for two or more hours on weekdays, and the prevalence increases with age [2]. Furthermore, results of the EU Kids Online II survey [3] indicate that 9- to 16-year-old children use the Internet almost an hour and a half every day, and this increases steeply with age. It is worth mentioning that one of the most important online activities is playing games, which was reported by 83% of European children in the EU Kids Online II survey [3].

One of the factors also contributing to an increased amount of time spent using media might be the availability of specific electronic devices, such as a TV or computer, in the bedroom [4, 5]. Research suggests that around half of 12- to 15-year-old adolescents have a TV in their bedroom [6], and these children, especially younger children, spend approximately 20–30 minutes more time in front of the TV per day than those without a TV in their bedroom [7].

The family is the primary social environment that influences the behaviour of children and adolescents. The presence of parental rules focused on limiting screen time appeared to be one of the key factors that influence the amount of time spent on screen-based behaviour in youth [8]. Data shows that children whose parents report low restrictions on sedentary activities were most likely to watch TV even more than four hours per day. Moreover, a permissive parenting style is associated with increasing risk of such excessive TV viewing among younger adolescents [9]. Similar results for playing on a computer have been found in other studies. Adolescents who exceeded the recommended screen time, had no parental screen-viewing rules [10] or who lacked parental restriction on Internet use were more likely to report an increased amount of time spent online [11]. On the other hand, studies are not consistent about the link between parental rules regarding time spent on screen-based activities and the number of hours children use computers. For instance, Sook-Jung and Young-Gil [12] found no effect of various parental restrictions (e.g. time limits, web site restriction) on children’s online activities, including playing games; however, time spent with online activities was not considered. Therefore, more research is needed to understand the role of parents in moderating children’s screen-based activities.

Research suggests that excessive time spent on screen-based activities among adolescents is also associated with the activities of their parents. Some studies show that families are most often engaged with one another during leisure-time activities or eating meals together [13, 14]. Findings indicate that family TV viewing, as a part of a family’s time spent together, is associated with increased television time, especially among older adolescents [15, 16].

In addition, parents influence the amount of time children spend using media in another way. The mean time children spend on leisure activities is mainly structured by parents and particularly by the extent to which parents share activities with their children [17]. In relation to computer use and playing computer games, a recent literature review suggests that excessive time spent on screen-based activities in children may be an outcome from the lack of unstructured spare time [18]. Concerning TV use, findings indicate that family TV viewing, as a part of a family’s time spent together, is associated with increased television time, especially among older adolescents [15, 16].

Countries like Slovakia or the Czech Republic are of specific interest, particularly due to the current rapid changes in the prevalence and patterns of screen-based activities that might be envisaged. According to a report of the EU Kids Online survey [19], e.g. in 2006 Slovakia and the Czech Republic were lagging behind other European countries in terms of Internet connectivity at home, but since that time a dramatic change has taken place in this field, which might be related to changes in time spent on screen-based activities as well.

The expansion of electronic devices into many European societies has changed what children do in their leisure time. For instance, it has been shown that the prevalence of extensive time spent on screen-based activities, such as playing games, is increasing in Slovak as well as in Czech children [11, 20], which is in turn related to physical and psychological health complaints [21–23]. A high level of sedentary behaviour was related to sleep problems and musculoskeletal pain [22, 24]. Spending a high number of hours on a computer was related to neck pain [25], as well as to recurrent backache and headache [22, 26]. More sedentary time of any type was associated with more psychological complaints, such as depression, or those related to well-being, social support [24] or poorer self-esteem [27]. To understand these associations with spending time in front of screens and to prevent the onset of potential health problems, it is important to consider family-related factors which might play an important role in moderating the extensive time spent on screen-based activities by these children.

The aim of this study is to explore the association between family-related factors (the availability of a TV or computer in the bedroom, parental rules on time spent watching TV or on computer use and the content of such TV watching or computer work, and activities spent together with parents) and excessive time spent on screen-based activities among school-aged children.

## Methods

### Sample and procedure

This study is based on the international Health Behaviour in School-aged Children (HBSC) study and is consistent with its methodology. The HBSC is an international, school-based study conducted in collaboration with the World Health Organization and focusing on the health and health-related behaviour of 11-, 13- and 15-year-old school children in their social context. More detailed information about the HBSC methodology can be found in a paper by Roberts et al. [28].

The study was conducted in November 2013 in the Czech Republic and Slovakia and was preceded by a pilot study which included the administration of questionnaires and the use of focus groups in both countries. Based on the data obtained in the pilot study the final set of questions was compiled. We contacted 16 larger and smaller primary schools located in rural as well as in urban areas in the Olomouc region, Czech Republic (7 schools), and the Kosice region, Slovakia (9 schools). The prevalences regarding the explored variables (e.g. family structure, screen-based behaviour, parental rules) in the recruited samples are rather similar to those in other studies covering all regions, so we anticipate that our findings on the associations between family-related factors and adolescent screen-based activities of the adolescent population in the Czech and Slovak republics can be generalized to a wider sample. The schools were randomly chosen to create a representative sample. We succeeded in achieving a 100% response rate on the school level, since all of the contacted schools agreed to participate. Questionnaires were administrated in the 5th and 9th grades by trained research assistants in the absence of a teacher during regular class time.

We obtained data from 906 adolescents in the Czech Republic (response rate: 83.20%) and Slovakia (response rate: 74.14%). Non-response was primarily due to illness and parental non-consent regarding the participation of their children. The final sample consisted of 418 Czech (46.1% boys) and 488 Slovak (53.9% boys) primary school pupils, grades five (mean age 10.93, SD = 0.62) and nine (mean age 14.90, SD = 0.44).

The study was approved by the Ethics Committee of the Faculty of Physical Culture, Palacky University in Olomouc (decision from May 15^th^ 2013) and by the Ethics Committee of the Medical Faculty at P J Safarik University in Kosice (decision from June 18^th^ 2012). The schools selected in the Czech Republic have a general permission granted at the beginning of the school year by all parents. Parents in Slovakia were informed about the study via the school administration and could opt out if they disagreed with it. Participation in the study was fully voluntary and anonymous with no explicit incentives provided for participation in either country.

### Measures

**Screen-based activities**, represented by watching TV and playing computer games, were assessed using two separate items. Watching TV was measured by the question: “About how many hours a day do you usually watch television (including videos) in your free time?” Computer gaming was measured by asking: “About how many hours a day do you play PC-games or TV-games (PlayStation, Xbox, GameCube etc.) in your free time?” All questions had the same nine response categories separately for weekdays and weekends: None at all, About half an hour a day, About 1 hour a day, About 2 hours a day, About 3 hours a day, About 4 hours a day, About 5 hours a day, About 6 hours a day, About 7 or more hours a day [26].

Using recommendations of the American Academy of Pediatrics [29], the categories of excessive and non-excessive time spent on screen-based activities were created by dichotomizing responses into two groups: those who spent less than 2 hours per day and those who spent 2 or more hours per day on screen-based activities.

The availability of a **TV or computer in the bedroom** was assessed using a single item asking adolescents if they have the following things in their bedroom, where they sleep and study: radio/CD player, TV, computer, Internet. Each electronic device had two categories of responses: yes or no. The responses used in the statistical analyses referred to a TV and a computer.

Family structure was measured by asking respondents the following question: “The family, where you live is … (1) a two-parent household, neither is a step-parent (2) one parent is a step-parent, (3) a single-parent household.

**Parental rules** were items focused on restrictions related to TV or computer use based on previous studies [8, 15, 30, 31]. To assess the objective of our study, we used separate items related to limitation of the time and content of TV programmes and computer work; adolescents were asked to indicate to what extent selected rules were applied in their family. Respondents were asked to answer the following questions: “My parents limit the time spent with watching TV.”; “My parents limit the content of the programmes I watch in TV.”; “My parents limit the time spent with playing PC games.”; “My parents limit the content of PC work.” Responses were on a 4-point scale: always, mostly, rarely, never and were dichotomized into rarely and never, vs. almost every day and every day.

**Family activities** were evaluated on a scale adopted from Sweeting et al. [32] and were assessed using a list of eight activities which some families commonly do together. Participants indicated how often they and their family usually do each of the shared activities together, including watching TV or a video, playing indoor games, eating a meal, going for a walk, going places, visiting friends or relatives, playing sports and sitting and talking about things [33]. Responses for frequency were on a 5-point scale: everyday, most days, about once a week, less often and never. Since all these activities – with the exception of watching TV together – are indicators of a latent variable which may be described as “family team spirit”, we used principal component analysis to extract one factor representing this latent variable. All of the above-mentioned variables were entered into this factor with almost the same weight, making it easily interpretable. This factor explains about 50% of the cumulative variance. We constructed a variable representing all shared family activities, but excluding watching TV together with parents; the latter we used as a separate variable. Responses were dichotomized into two categories as follows: (1) watching TV together every day, (2) watching TV together on most days, about once a week, less often and never.

### Statistical analyses

Country, gender and age differences in family-related factors as well as screen-based behaviour variables were explored using the chi-square for dichotomous variables and the t-test for independent variables for continuous variables. The association between family-related factors of excessive time spent watching TV and playing computer games was explored using logistic regression models. Firstly, each variable was entered separately into the model adjusted to age and gender (Model 1). When age and gender differences were confirmed in exposure variables, interactions were considered in analytical models. Each interaction was included separately into the model (Model 2). Finally, variables which significantly contributed to the prediction of the outcome measure were included into the model in one step (Model 3).

## Results

Two-thirds of respondents watched TV or played computer games at least two hours a day (see Table 1). Czech children did not differ from Slovak children (TV/PC: chi square =0.319/1.355, ns/ns); therefore, we did not consider this variable in further models.

**Table 1:** **Descriptive characteristics of the sample**

		N (%)
**Country**	Slovak Republic	488 (53.9)
	Czech Republic	418 (46.1)
**Gender**	Boys	478 (52.8)
	Girls	428 (47.2)
**Age (grade)**	11-years old (5th grade)	458 (50.6)
	15-years old (9th grade)	448 (49.4)
**TV located in bedroom**	Yes	470 (52.6)
	No	424 (47.4)
**PC located in bedroom**	Yes	661 (73.4)
	No	240 (26.6)
**Family completeness**	Complete intact family	704 (80.1)
	Complete mixed family	103 (11.7)
	Incomplete family	72 (8.2)
**Parents apply rules about time spent watching TV**	Rarely-never	518 (63.9)
	Every day-almost every day	293 (36.1)
**Parents apply rules about content of TV programmes**	Rarely-never	563 (69.6)
	Every day-almost every day	246 (30.4)
**Watching TV together with parents**	Every day	226 (25.3)
	Most days	666 (74.7)
**Parents apply rules about time spent with a computer**	Rarely-never	446 (55.5)
	Every day-almost every day	357 (44.5)
**Parents apply rules about the content of computer work**	Rarely-never	531 (66.8)
	Every day-almost every day	264 (33.2)
**Watching TV**	Less than 2 hours a day	305 (36.0)
	2 or more hours a day	543 (64.0)
**Computer use**	Less than 2 hours a day	340 (40.2)
	2 or more hours a day	506 (59.8)

### Association between family-related factors and excessive time spent watching TV

Children who were 15 years old who had a TV located in their bedroom, whose parents rarely or never applied rules on the length of TV watching and who watched TV together with parents every day had a significantly higher chance of spending excessive time watching TV. Family completeness or applying rules on the content of TV programmes watched do not contribute significantly to the prediction of excessive watching TV (Model 1).

The effect of either or both parental rules and activities shared with parents on the chances of spending excessive time watching TV does not differ between children aged 11 and 15 years old (see Table 2, model 2).

**Table 2:** **The associations of factors of family context with excessive spending of time watching TV among adolescents**

			Watching TV 2 hours and more		
			Model 1	Model 2	Model 3
		N (%)	OR (95% CI)		OR (95% CI)
**Gender**	Boys	293 (66.1)			1.18 (0.87-1.61)
	Girls	250 (62.0)			1
**Age (grade)**	15-years old (9th grade)	324 (73.3)			1.80 (1.30-2.51)***
	11-years old (5th grade)	219 (53.9)			1
**Family completeness**	Incomplete	51 (72.9)	1.46 (0.83-2.57)		
	Mixed	67 (67.7)	1.15 (0.72-1.83)		
	Intact	411 (62.8)	1		
**TV located in bedroom**	Yes	305 (70.1)	1.64 (1.23-2.20)**		1.59 (1.17-2.16)**
	No	233 (57.8)	1		1
**Parents apply rules about time spent watching TV**	Rarely-never	370 (71.7)	1.84 (1.33-2.56)***		1.76 (1.26-2.46)**
	Every day-almost every day	153 (53.1)	1	^a^	1
**Parents apply rules about content of TV programmes**	Rarely-never	389 (69.2)	1.40 (0.99-1.97)		
	Every day-almost every day	132 (54.8)	1	^b^	
**Watching TV together with parents**	Every day	157 (74.8)	1.95 (1.36-2.79)***		1.84 (1.25-2.70)**
	Most days	381 (60.8)	1		1
**Activities shared with parents**	Lower score = more frequently shared activities		0.99 (0.85-1.17)	^c^	

^a^the interaction effect of parental rules about time spent watching TV and age on chance of excessive TV watching was not significant.

^b^the interaction effect of parental rules about the content of TV programmes and age on chance of excessive TV watching was not significant.

^c^the interaction effect of activities shared with parents and age on chance of excessive TV watching was not significant.

Model 1 Each variable separated and adjusted to age and gender.

Model 2 Model 1 enriched by interaction.

Model 3 All variables included in one step.

**p < 0.05 **p < 0.01 ***p < 0.001.

The effect on excessive TV watching of age, having a TV in the bedroom, applying rules on time spent watching TV or the content of TV programmes watched, as well watching TV with parents, remained significant also in the adjusted model (see Table 2, model 3). Children aged 15 years old have a 1.80-times higher chance of excessive TV watching in comparison with 11-year-old children. Having a TV located in the bedroom increases the chances of excessive TV watching by 1.59 times, and children reporting that their parents rarely or never apply rules on time spent watching TV have a 1.76-times higher chance of excessive TV watching. Children who watch TV with their parents every day have a 1.84-times higher chance of excessive TV watching.

### Association of family-related factors and excessive time spent playing computer games

Boys, children aged 15 years old who had a computer available in their bedroom or whose parents rarely or never apply rules on the length of computer work had a significantly higher chance of spending excessive time playing computer games. Completeness of the family, applying rules on the content of computer work and activities shared with parents do not contribute significantly to the prediction of excessive computer use (see Table 3, model 1).

**Table 3:** **The associations of factors of family context with excessive spending of time on computer use among adolescents**

			Work with computer 2 hours and more		
			Model 1	Model 2	Model3
		N (%)	OR (95% CI)		OR (95% CI)
**Gender**	Boys	289 (65.4)			1.62 (1.18-2.22)**
	Girls	216 (53.7)			1
**Age (grade)**	15-years old (9th grade)	341 (77.5)			3.91 (2.82-5.43)***
	11-years old (5th grade)	165 (40.6)			1
**Family completeness**	Incomplete	45 (66.2)	1.16 (0.66-2.05)		
	Mixed	58 (58.6)	0.75 (0.47-1.21)		
	Intact	392 (60.0)	1		
**Computer in bedroom**	Yes	411 (67.2)	2.42 (1.73-3.39)***		2.25 (1.59-3.20)***
	No	94 (40.7)	1	^a^	1
**Parents apply rules about spending time with a computer**	Rarely-never	310 (69.8)	1.65 (1.19-2.28)*		1.50 (1.07-2.08)*
	Every day-almost every day	175 (49.4)	1	^b, c^	1
**Parents apply rules about content of computer work**	Rarely-never	355 (67.2)	1.27 (0.90-1.80)		
	Every day-almost every day	124 (47.5)	1	^d^	
**Activities shared with parents**	Lower score = more frequently shared activities		0.97 (0.82-1.15)	^e^	

^a^the interaction effect of the computer in the bedroom and age on the chance of excessive computer use was not significant.

^b^the interaction effect of parental rules about time spent with computer work and age on chance of excessive computer use was not significant.

^c^the interaction effect of parental rules about time spent with computer work and gender on chance of excessive computer use was not significant.

^d^the interaction effect of parental rules about content of computer work and age on chance of excessive computer use was not significant.

^e^the interaction effect of activities shared with parents and age on chance of excessive computer use was not significant.

Model 1 Each variable separated and adjusted to age and gender.

Model 2 Model 1 enriched by interaction. Each interaction entered separately.

Model 3 All variables included in one step.

**p < 0.05 **p < 0.01 ***p < 0.001.

The effect of having a computer available in the bedroom or any of the parental rules and activities shared with parents on the chance of spending excessive time playing computer games did not differ between 11-year-old and 15-year-old children (see Table 3, model 2). Similarly, the effect of parental rules concerning time spent with a computer on the excessive use of a computer did not differ between boys and girls.

The effect of gender, age, having a computer located in the bedroom and applying parental rules on time spent with a computer on excessive computer use remained significant also in the adjusted model (see Table 3, model 3). Children aged 15 years old had a nearly four-fold higher chance of spending excessive time playing computer games in comparison with younger children, and boys had a 1.62-times higher chance of spending excessive time playing computer games than girls. Having a computer located in the bedroom increased the chance of spending excessive time watching TV by 2.25 times, and children reporting parental rules rarely or never on time spent playing computer games had a 1.50-times higher chance of spending excessive time playing computer games.

## Discussion

Our results showed that parental rules that restrict the time spent on screen-based activities were associated with a lower probability of excessive spending time on screen-based activities, but that parental rules restricting the content of TV programmes or computer work were not. This result supports previous findings from the existing body of research showing that what parents do regarding children and their media use matters [10, 11]. Although restrictions on the content of screen-based activities are not relevant for the amount of time spent, they may still be relevant for the content of screen-based activities. We did not aim to identify which content gives the highest risk of excessive use (e.g., soap operas, long-lasting PC games). In connection with risk factors, Blinka et al. [34] showed that playing online games is a risk factor associated with highly excessive Internet use. Given these results we can conclude that this issue requires further study with more detailed questions than those we used in this study.

In line with prior research [15, 16], we found that Czech and Slovak children who shared watching TV with their parents every day were more likely to report excessive TV watching. An explanation may be that watching TV with parents does not give children another model of how to spend their leisure time; it does not promote adequate structuring of their leisure time, so the association with excessive TV watching is not surprising.

Our results showed that one of the most important predictors of excessive time spent on screen-based activities was having either a TV or a computer in the bedroom. The association of a higher number of screens in a child’s bedroom and more total screen time has also been indicated in other studies [35]. Generally, it is today common that children grow up in bedrooms equipped with various media devices. This phenomenon has been described in detail in the theory of the “bedroom culture”. According to the concept, children spend less time in the streets or on playgrounds in favour of staying at home in their media-rich bedrooms, which provide them with a large scale of stimulations [4].

This study has several strengths, but it also has some limitations. An important strength is the international HBSC methodology used and the fact that the obtained data are from two Central European countries. On the other hand, the main limitation is that self-reported data were used, and we do not have available data from parents that would confirm the responses of their children. However, self-reporting has been previously shown to offer satisfying reliability in terms of health-related behaviour [36]. Another limitation is the cross-sectional design of this study, which made it impossible to formulate conclusive statements about causality. Our findings, therefore, need to be confirmed in longitudinal studies. Moreover, in our study we did not include the use of smartphones, which are a highly portable electronic device that can also be combined with physical activities and outdoor/indoor use. In contrast, typical/classic screen-based activities tend to naturally disrupt physical activities and are limited to indoor use [37]. Therefore, the effect of smartphone use on physical activity deserves further study.

## Conclusions

More and more school-aged children spend excessive time on screen-based activities, and the number of these children increases every year, mainly among older peers. It seems that there are several factors which could have an important impact on screen time. The availability of specific electronic devices in their own bedrooms, for example, or the fact that parents or even family-shared activities might play key role in reducing excessive time spent in front of screens. Based on our results, parents and applying rules about watching TV or playing computer games have only a moderate effect. The home environment and parents may still partly influence the behaviour of their children, including how they spend their leisure time. They could keep in mind that they have an opportunity to change the “unhealthy” lifestyle of their children and encourage them to do alternative activities which could be more beneficial to them.
